# Clinical use of bone markers: a challenge to variability

**DOI:** 10.1515/almed-2023-0092

**Published:** 2023-08-28

**Authors:** Xavier Filella, Núria Guañabens

**Affiliations:** Servicio de Bioquímica y Genética Molecular (CDB), Hospital Clinic, IDIBAPS, Barcelona, Spain; Servicio de Reumatología, Hospital Clinic, IDIBAPS, Universitat de Barcelona, Barcelona, Spain

**Keywords:** bone markers, CTX, PINP

## Abstract

Bone markers are a group of substances released into circulation during bone formation and/or resorption. These substances can be measured in blood and urine to obtain information about metabolic bone disorders. This review provides an insight into factors influencing bone marker variability and describes different approaches to minimize variability and interpret results appropriately. Variability in bone marker concentrations results from biological and analytical variability across assays. Other influencing factors include gender, age, physical exercise, circadian rhythm, and diet. The multiplicity of influencing factors hinders the establishment of accurate reference values. Gaining a deep understanding of bone marker variability is the first step to ascertain their clinical usefulness. Bone marker variability can be minimized by controlling as many variables as it is possible and through the standardization of patient preparation and sample collection and handling.

## Introduction

Bone markers (Table 1) are a group of substances released into circulation during bone formation and/or resorption. These substances can be measured in blood and urine to obtain information about metabolic bone disorders [1, 2]. Bone markers are not useful for the diagnosis of osteoporosis and cannot be used instead of bone density scanning to measure bone mass density. However, bone markers identify patients at a higher risk of fracture, when considered in relation to other risk factors, especially for the early assessment of response to antiresorptive agents or anabolic steroids. Bone markers have been used for follow up in other bone disorders, including Paget’s bone disease, hyperparathyroidism, osteomalacia, renal osteodystrophy and bone metastases.

**Table 1:** Description of relevant bone markers.

Acronym	Name	Metabolic process	Source	Type of sample	Method	Automatization (analyzer)
CTX	Collagen type I β-isomerized C-terminal telopeptide	Resorption	Bone (and other tissue containing type I collagen)	Serum/plasma (urine)	ELISA, RIA, electrochemiluminescence, chemiluminescence	Yes (Cobas, IDS-iSYS)
ALP	Bone alkaline phosphatase	Formation	Osteoblasts	Serum/plasma	Electrophoresis, colorimetry, ELISA, RIA, chemiluminescence	Yes (Liaison, Access/UniCel DxI, IDS-iSYS)
NTX	Collagen type I N-terminal telopeptide	Resorption	Bone (and other tissue containing type I collagen)	Urine	ELISA, RIA, chemiluminescence	Yes (Vitros)
OC	Osteocalcin	Formation	Osteoblasts, dentine, hypertrophic cartilage	Serum/plasma (urine)	ELISA, RIA, electrochemiluminescence, chemiluminescence	Yes (Cobas, IDS-iSYS, Immulite, Liaison)
PINP	Procollagen type I N-terminal propeptide	Formation	Bone (and other tissue containing type I collagen)	Serum/plasma	ELISA, RIA, chemiluminescence	Yes (Cobas, IDS-iSYS)
SC	Sclerotine	Inhibits bone formation	Osteocytes, chondrocytes	Serum/plasma	ELISA, chemiluminescence	Yes (Liaison)
TRAP-5b	Tartrate-resistant acid phosphatase	Resorption	Osteoclasts	Serum/plasma	Colorimetry, RIA, ELISA, chemiluminescence	Yes (IDS-iSYS)
FGF-23	Fibroblast growth factor 23	Proximal phosphate reabsorption Reduction of 1,25 OH vitamin D and PTH	Osteocytes	Plasma	ELISA, chemiluminescence	Yes (Liaison)

For a correct clinical interpretation of bone marker concentrations, variability should be considered [3]. The term variability embraces analytical variability resulting from assays, and pre-analytical variability caused by a range of influencing factors, including gender, age, circadian rhythm, physical exercise, a recent fracture, or diet. The lack of standardization in the different commercially-available assays for measuring bone marker concentrations hampers their clinical use. Gaining a deep understanding of bone marker variability is the first step to ascertain their clinical usefulness.

In 2010, the International Osteoporosis Foundation (IOF) and the International Federation of Clinical Chemistry and Laboratory Medicine (IFCC) recommended measuring procollagen type I N-terminal propeptide (PINP) as a marker of bone formation; and collagen type I β-isomerized C-terminal telopeptide (CTX) as a marker of bone resorption [4]. PINP is the product of the action of specific proteinases, by which type I procollagen is synthetized to type I collagen by fibroblasts and osteoblasts. CTX results from type I collagen degradation by a diversity of proteases released by osteoclasts during bone resorption.

PINP and CTX have been used in clinical trials on osteoporosis and for patient management. These markers are frequently selected owing to the availability of scientific evidence, understanding of their biological and analytical variability, easy handling of samples, analyte stability, assay simplicity, and the fact that they are measured in serum instead of urine. There are assays currently available that measure PINP and CTX on IDS-iSYS (Immunodiagnostic Systems) and Cobas (Roche) analyzers. Some differences arise depending on whether PINP is determined on one or another analyzer. There are two circulating forms of PINP, the intact – or trimeric – and the monomeric form. Whereas the IDS-iSYS assay only measures intact PINP, the Cobas assay measures both, the trimeric and the monomeric form (total PINP). This difference is relevant in patients with kidney diseases, since monomeric PINP is affected by renal insufficiency, whereas trimeric PINP is not influenced by changes in renal function. Intact PINP can be also measured by radioimmunoassay (Orion Diagnostica), whereas total PINP can be determined by ELISA (USCN Life Science Inc.).

Alike CTX, procollagen type I N-terminal propeptide (NTX) is a product of type I collagen degradation, although it corresponds to the N-terminal fragment. NTX can be measured in urine by ELISA or by chemiluminescence on a Vitros analyzer (Ortho Clinical Diagnostics). For such purpose, a second-morning urine sample should be collected. Urine contaminated with blood must be disposed of due to interference. Urine NTX results are expressed in relation to creatinine concentrations in the sample. As a result, measurement is influenced by analytical variability in the two analytes.

There are specific monoclonal antibodies that enable the measurement of bone alkaline phosphatase and tartrate-resistant acid phosphatase 5b (TRAP-5b). The former reflects osteoblast activity, and the latter indirectly indicates the number of osteoclasts. Bone alkaline phosphatase measurement is useful for the management of patients with Paget’s disease or osteoporosis, with low cross-reactivity with other alkaline phosphatase isoenzymes. TRAP-5b is a tartrate-resistant isoform of the acid phosphatase family that, unlike the 5a fraction present in macrophages, is expressed in osteoclasts.

Apart from manual ELISA assays, there are automated assays that measure the two markers on the IDS-iSYS analyzer, whereas bone alkaline phosphatase can be measured on the automated Liaison (Diasorin), Access and Unicell DxI (Beckman Coulter) analyzers. The availability of immunoassays that measure the two markers has made it possible to overcome many of the drawbacks of other techniques, such as electrophoresis or colorimetry, which have been used until a few years ago.

Osteocalcin, produced by osteoblasts, is the most important non-collagen protein of the bone matrix. Apart from bone formation, osteocalcin regulates energy metabolism by acting on beta-pancreatic cells and adipocytes, thereby providing the energy required for bone remodeling. In the past, osteocalcin was frequently used as a marker of bone formation. However, this marker is currently used only for some indications; for example, osteocalcin is used as a predictor of the risk of developing diabetes mellitus in glucocorticoid-treated patients or for monitoring glucocorticoid-induced osteoporosis and associated chronic cholestasis [5]. Blood contains both, intact osteocalcin (aminoacids 1–49) and the N-terminal midfragment (N-mid) (aminoacids 1–43), with the latter being considerably more stable. Most of the assays available for the measurement of osteocalcin, many of which are performed on automated analyzers (Cobas, IDS-iSYS, Immulite, Liaison) determine the intact molecule and the N-mid fragment.

Sclerostin, secreted by osteocytes, is one of the main regulators of bone formation, albeit it is not a marker of bone turnover. An increase or decrease of its activity is linked to diseases presenting with osteoporosis or osteosclerosis, respectively. Its measurement is especially useful in patients with severe osteoporosis. An automated assay has recently become available for use on the Liaison analyzer, although sclerostin can also be measured by ELISA.

The fibroblast growth factor 23 (FGF-23) belongs to the phosphatonin group and is synthetized by the osteocyte. FGF-23 inhibits proximal phosphate reabsorption by blocking PTH synthesis and release, and reduces 1,25 OH vitamin D concentrations through the inhibition of renal 1α-hydroxylase. FGF-23 activity is mediated by its binding with the signaling complex composed of FGF receptor and α-Klotho, a protein that increases FGF receptor affinity for FGF-23. A variety of ELISA assays measure the biologically active intact form of FGF-23 (including Immunotopics and Kainos), whereas the C-terminal fraction is measured by ELISA (Immunotopics). Since 2017, an automated Liaison assay is available, which measures intact FGF-23 and is CE-certified.

## Biological variability

Biological variability, which is essential for the correct interpretation of a biomarker, includes within-subject and between-subject biological variability. Within-subject variability results from physiological fluctuations around repeated measures in the same individual. Between-subject variability results from variability of a given biomarker across different individuals.

Measuring biological variability is important when it comes to determine whether a change in a marker is or not a clinically significant response. The cut-off value of a change in the concentration of a biomarker, also known as a minimal significant change, is derived from the estimation of analytical variability added to within-subject biological variability. Measuring biomarker concentration change is useful for monitoring response to antiresorptive or anabolic agents and estimating treatment adherence, which are the main indications for measuring bone marker concentrations.

It has long been known that biological variability in bone markers is induced by factors that influence bone remodeling including, but not limited to, humoral regulation, diet and physical activity [6, 7]. The biological variability database of the European Federation of Clinical Chemistry and Laboratory Medicine (EFLM) [8] provides the body of evidence available on biological variability in different analytes. However, according to the biological variability data critical appraisal checklist (BIVAC), most studies provide low quality evidence (C degree).

The EFLM Working Group on Biological Variability conducted a study to overcome this drawback. In this study, samples of fasting EDTA plasma were serially collected from 38 men and 53 women over a 10-week period. Thus, a sample was collected weekly at 8–10 a.m. The study revealed a within-subject biological variability of 15.1 % (CI 95 %: 14.4–16 %) for CTX, being lower for PINP (8.8 %; 95 % CI: 8.4–9.3 %) and osteocalcin (8.9 %; 95 % CI: 8.5–9.4 %) [9]. On another note, the Adherence Working Group of the IOF and the European Calcified Tissue Society recommends measuring CTX and PINP at baseline and at three months from initiation of antiresorptive therapy. Minimal significant change was a decrease of more than 56 % for CTX and 38 % for PINP [10].

Regarding NTX, Eastell et al. [11] established a short-term within-subject variability of 13.1 % (with samples collected over three consecutive days) for urinary NTX, and a long-term within-subject variability of 15.6 % (with samples collected for two months following baseline sample collection). According to the authors, for a decrease in NTX after an antiresorptive therapy to be considered minimally significant, it should exceed 31 %.

According to the EFLM Working group on biological variability, within-subject biological variability of intact FGF-23 is 13.9 % (95 % CI: 13.2–14.7) [9]. In contrast, Smith et al. [12] document a lower within-subject variability in the C-terminal fraction of FGF-23, as compared to that of intact FGF-23 (8.3 vs. 18.3 %).

A lower biological variability is reported for other bone markers. Álvarez et al*.* [7] documented a within-subject variability of 3.4 % for bone alkaline phosphatase in healthy subjects, and 4.9 % in patients with Paget’s disease. Finally, the EFLM Biological Variability database indicates a within-subject variability of 6.6 % for bone alkaline phosphatase and 10.8 % for tartrate-resistant acid phosphatase [8].

## Analytical variability

Analytical variability should also be considered, in terms of between-assay and within-assay variability, being lower in automated assays, as compared to manual assays. Variability also occurs across the different commercially available assays, since a gold standard measurement method has not yet been established, and reference materials are not available for calibration.

The IFCC-IOF Joint Committee for Bone Metabolism assessed the level of harmonization of PINP assays. The study revealed that IDS-iSYS and Cobas yielded very similar results when glomerular filtration rate was >30 mL/min/1.73 m^2^, but with a proportional bias between Orion RIA and the automated methods for PINP [13]. In the same line, Guañabens et al*.* [14] reported a small bias between automated PINP assays on IDS-iSYS and Cobas in pre-menopausal women. However, these authors report a higher bias between the two analyzers when measuring CTX. The position of the IFCC-IOF Committee for Bone Metabolism is in agreement with that of Guañabens. However, the Committee points out that differences increase when measurement is performed in serum. Therefore, the Committee recommends the use of plasma EDTA for measuring CTX, especially in studies involving the use of frozen specimens [15].

These inconsistencies hamper comparison of results across studies when different assays have been used to measure a specific analyte. This situation hinders the harmonization of reference values for clinical decision-making. Also, it makes it more difficult to leverage the accumulated clinical experience with different reagents. To overcome this drawback, the IFCC-IOF Committee for Bone Metabolism proposed preparing commutable international reference materials and developing standard measurement procedures for PINP and CTX in blood. This initiative would facilitate the establishment of standard reference intervals and universally acceptable limits for clinical decision-making [16].

The deficient generalizability of results across the different commercially available assays also affects other markers. Schafer et al. [17] found inconsistencies in the measurement of NTX between the Osteomark assay and Vitros. Eastell et al*.* [18] reported substantial differences in the determination of bone alkaline phosphatase between the Metra BAP EIA test and the automated Access assay. Likewise, there are notable differences in the intervals of reference recommended by the manufacturers of the different assays for measuring osteocalcin, although most measure both, intact osteocalcin and the N-mid fragment [19]. Differences have also been documented across sclerotin assays, having different reference intervals [20]. The different FGF-23 assays also show inconsistencies, and results are expressed in different units [21].

## Variability in the definition of the reference interval for bone markers

Establishing reference intervals for the different bone markers is essential for an appropriate clinical use. Firstly, because the aim of antiresorptive therapy with bisphosphonates is to reduce levels of bone marker concentrations to the lower half of the reference interval established for pre-menopausal women. Secondly, the availability of cut-off values would help determine whether bone markers, when used in combination with other factors, can be used as predictors of the risk for fracture.

However, despite their relevance, robust reference intervals are not available, with occasionally-significant inconsistencies even when measurements are performed using the same assay. The availability of standardized assays that yield comparable results will enable the establishment of robust reference values. For that purpose, multicenter studies involving an optimal patient selection are necessary, although some studies have already been carried out [14, 18, 22].

The reference interval established for women is based on concentrations in premenopausal women, who have a low bone turnover and an optimal bone health. In men, the reference intervals have been established on the basis of concentrations measured in men aged 40–60 years [2].

Differences in reference intervals are more substantial in CTX than in PINP. The upper and lower limit for CTX reported by Morris et al*.* [23] for premenopausal women, as measured on a Cobas analyzer, range from 70 to 163 ng/L and from 274 to 800 ng/L, respectively. Differences are less substantial for PINP, as the lower limit ranges from 14.6 to 22.7 μg/L, and the upper limit from 42.9 to 90 μg/L.

Schini et al*.* [2] established the lower limit for CTX in premenopausal women at 70–163 ng/L, and the upper limit at 274–895 ng/L. In contrast, differences in the lower and upper limits were less significant in PINP, ranging from 13.72 to 22.7 μg/L and from 42.9 to 99 μg/L, respectively. The same authors report a lower limit of 93–252 ng/L, and an upper limit of 400–1,116 ng/L for CTX. In the case of PINP, the differential range for the lower and upper limits was 16.9–29.4 μg/L and 43.9–98 μg/L, respectively.

Of note, Glover et al. [22] assessed geographical differences in reference intervals for bone markers. The authors measured PINP, CTX, NTX and bone alkaline phosphatase in 637 healthy premenopausal women with regular cyclic menstruations living in the United Kingdom, France, Belgium and the United States. The authors found no differences in urinary NTX and bone alkaline phosphatase concentrations. In contrast, differences were observed in PINP and CTX. CTX was significantly higher in France, as compared to UK. PIPN was higher in France and Belgium, as compared to UK, and in France with respect to USA. Significant differences were observed among women from different countries, in terms of body mass index, weight, previous pregnancies, 25-OH vitamin D, smoking habits, regular physical exercise and alcohol use. Differences in PINP and CTX concentrations across the four populations are probably explained by the effects of these factors.

Age- and gender-based differences are consistently reported, with concentrations being higher in men than in women, and in older adults, as compared to young patients. Concentrations are significantly higher during childhood. An L pattern has been observed, with bone marker concentrations decreasing slightly between 30 and 35 years, and remaining stable between 35 and 45 years [13, 24].

Adami et al. [25] provided evidence of the difficulty in establishing reference intervals for bone markers. The authors measured PINP, CTX and osteocalcin in 638 premenopausal women aged 20–50 years categorized into six age groups. The study confirms the L pattern in bone marker concentrations and their stabilization in women aged 35–50 years. The study also reveals that there is a subgroup of women with FSH concentrations >30 IU/mL with significantly higher PINP, CTX and osteocalcin concentrations, despite having regular cyclic menstruations.

Therefore, there are inconsistencies in the reference intervals provided across studies. It is worth noting the data provided by Eastell et al*.* [18] in a study conducted to determine a reference interval for CTX, PINP, NTX and bone alkaline phosphatase in a population of 194 French and Danish premenopausal women. The authors highlight the consistency of their results with another four previous studies.

## Factors contributing to variability in bone markers

A multiplicity of factors influence bone marker concentrations (Table 2). Their identification and control will improve the clinical use of bone markers. As described above, influencing factors include gender and age.

**Table 2:** Factors contributing to bone marker variability.

Factor	Effect
Circadian rhythm	Higher concentrations of resorption markers, especially CTX, at night, with lower concentrations in the afternoon. Osteocalcin follows a similar circadian rhythm
Seasonal rhythm	In general, concentrations are higher in osteocalcin, bone alkaline phosphatase and NTX in winter
Renal function	Dysfunction affects CTX, NTX and osteocalcin. Kidney function also has an impact on total PINP, but not on the intact form
Liver function	Liver function may affect CTX, NTX and PINP concentration
Age and gender	Higher concentrations in men than in women and in older adults with respect to young subjects, with concentrations being significantly higher during childhood, related to growth
Menopause	Bone turnover increases with the onset of menopause, with increased bone marker concentrations
Menstrual cycle	Lower bone resorption during the luteal phase
Pregnancy	During pregnancy, bone marker concentrations increase to progressively decrease after labor
Contraceptives	Influence bone marker concentrations, especially PINP, which concentration decreases
Physical exercise	Increased bone resorption with long-term immobilization. Intense physical exercise increases bone formation and reduces resorption
Food intake	Food intake hours before sample collection especially affects bone resorption-related markers
Diet and lifestyle	Dietary intake of calcium, vitamin D or calcium supplements, alcohol, smoking, and level of sedentarism

The circadian rhythm [26] is the first factor to be considered, as it will determine whether early morning sample collection is required. CTX is significantly influenced by circadian rhythm, with peak values occurring between 1:30 and 4:30 a.m., and minimum values occurring between 11 a.m. and 3 p.m. [2]. This fluctuation can be reduced if the sample is collected in fasting conditions at an established time. Osteocalcin also follows a circadian rhythm, and concentrations fall throughout the morning, increase at dusk, and peaks during the night [27]. Bollen et al*.* [28] observed that NTX index in relation to urinary creatinine is influenced by circadian rhythm. Thus, NTX decreases over the day, to reach a nadir in the late afternoon. FGF-23 also demonstrates circadian variability, especially the intact form. Thus, concentrations decline throughout the morning to reach a nadir at noon, then, it progressively increases to peak at midnight [29]. Therefore, it is recommended to draw blood between 8 and 10 a.m. Less significant circadian variability has been observed in PINP and bone alkaline phosphatase [26].

Seasonal changes also occur in bone marker concentrations. Variability could be explained by the increased bone turnover in winter, partially as a result of changes in vitamin D and PTH concentrations [30]. Higher concentrations have been documented in osteocalcin, bone alkaline phosphatase and NTX in winter [31], although the evidence provided in the literature is inconsistent.

On another note, liver function may influence CTX, NTX and PINP concentrations, whereas kidney function may affect CTX, NTX and osteocalcin concentrations [32]. Kidney function also has an impact on monomeric PINP, but not on the intact form. Bone alkaline phosphatase shows cross-reactivity with other isoenzymes, including hepatic isoenzymes, which could translate into higher concentrations in subjects with liver diseases.

Age-based differences have also been described in women, with significant variability in bone marker concentrations near menopause. The menstrual cycle also induces variability due to lower resorption during the luteal phase, although its impact is limited [33]. Contraceptives also influence bone marker concentrations, especially PINP [13]. During pregnancy, bone marker concentrations increase to progressively decrease over a year postpartum.

There is robust evidence about the impact of physical exercise on bone metabolism. Hence, long-term immobilization causes an increase in bone resorption, resulting in increased bone marker concentrations. A range of studies have been conducted to assess the influence of occasional and regular physical exercise on bone markers. However, results are not consistent [34].

There is evidence of variability in bone turnover induced by food intake. The mechanism by which food reduces bone resorption is not fully understood. Some authors suggest that it could be mediated with the release of intestinal and/or pancreatic peptides [35]. Clowes et al*.* [36] recommend that samples be collected in fasting conditions. Nevertheless, the authors remark that the intake of food has a limited impact, with the exception of CTX, with a 17.8 % variability reported between fasting and non-fasting samples.

Finally, diet and lifestyle may cause variability in bone marker concentrations, as demonstrated in the study by Glover et al*.* [23]. Some diet-related factors include the intake of calcium, vitamin D/calcium supplementation, alcohol use, smoking, or sedentarism.

A multicenter study was carried out in Catalonia, Spain, to assess the influence of a range of factors on CTX, NTX and PINP concentrations. Factors included age, body mass index, smoking habits, intake of calcium and alcohol, 25 OH vitamin D and PTH concentrations. Guañabens et al*.* [14] report a higher variability for CTX (with statistically significant differences based on age, body mass index, calcium intake, and PTH concentration), as compared to NTX (with statistically significant age-based differences only) or PINP (without any statistically significant differences having been observed).

Knowledge of factors influencing FGF-23 concentrations is more limited. Older adults exhibit higher FGF-23 concentrations, which could be associated with their lower Klotho concentrations. The association between FGF-23 and gender is unclear, although estrogens are known to reduce its concentrations. Finally, there is inconsistent evidence on the correlation between FGF-23 and ethnicity. Higher FGF-23 concentrations have been found in subjects with a low socioeconomic status, which would be associated with higher phosphate concentrations in these subjects [37].

On another note, it is worth mentioning that some conditions (inflammatory diseases, such as rheumatoid arthritis, liver diseases or conditions causing malabsorption such as celiac disease or Crohn’s disease), along with some therapies (Table 3) affect bone marker concentrations [2, 38, 39].

## Conclusions

Variability is the main challenge to a correct clinical use of bone markers. For this reason, it is necessary to control as many variables as possible (Table 4), with a standardized patient preparation and harmonized sample collection and handling. The stability of each bone marker should be considered in all cases (Table 5) [40].

**Table 3:** Treatments influencing bone markers.

Treatments	Effects on bone markers
Anabolics (Teriparatide)	Increase in bone formation markers, especially PINP. Resorption markers increase more slowly and with less magnitude
Antiresorptive agents	Rapid decrease of bone resorption markers, whereas bone formation markers decrease more slowly. They decrease more substantially in treatments with Denosumab, as compared to bisphosphonates
Antiandrogenic agents	Increase of remodelling-related markers
Vitamin K antagonists	Slight reduction of osteocalcin, but increase of uncarboxylated osteocalcin
Antiepileptic agents	Slight increase of remodelling-related markers
Contraceptives	They reduce bone marker concentrations, mainly bone formation markers, especially PINP
Glucocorticoids	Reduction of osteocalcin and PINP. Low influence on resorption-related markers, except in initial phases
Methotrexate	It reduces formation-related markers, including alkaline phosphatase and osteocalcin, and increase resorption-related markers
Calcium supplements	They reduce bone marker concentrations
Thiazides	Slight bone marker reduction

**Table 4:** General recommendations for sample collection.

– Patient preparation before sample collection: avoid strenuous exercise
– Collect the sample between 7:30 and 10:00 a.m. after 8 h fasting
– Collect second-morning urine if NTX is being measured, which will be expressed as a function of creatinine
– Consider analyte stability: bone alkaline phosphatase is very stable, PINP is more stable than CTX, osteocalcin is unstable
– Avoid hemolysis or the presence of blood in urine, it interferes with the measurement of osteocalcin and NTX
– Consider changes of assays for the measurement of a marker: perform comparative studies, inform clinical services in case of change

Firstly, sources of pre-analytical variability should be minimized. Appropriate patient preparation before sample collection should be ensured by asking the patient to avoid doing intense physical exercise the day before collection.

**Table 5:** Bone marker stability at room temperature and at 2–8 °C.

Acronym	Name	Stability at room temperature and at 2–8 °C
CTX	Collagen type I β-isomerized C-terminal telopeptide	At room temperature 8 h
		It can be stored at 2–8 °C for 8 h
		Higher stability in EDTA plasma: 24 h at room temperature and 8 days at 2–8 °C
ALP	Bone alkaline phosphatase	It can be stored at 2–8 °C for 3 days
NTX	Collagen type I N-terminal telopeptide	At room temperature 24 h
		It can be stored at 2–8 °C for 72 h
OC	Osteocalcin	At room temperature 1 h without serum separation and 8 h without EDTA plasma separation; 8 h after separation
		It can be stored at 2–8 °C for 72 h
PINP	Procollagen type I N-terminal propeptide	At room temperature 24 h
		It can be stored at 2–8 °C for 5 days
SC	Sclerotine	The sample must be centrifugated immediately, preferably at 2–8 °C
		Samples are stable at room temperature for up to 4 h
		It can be stored at 2–8 °C for 24 h
TRAP-5b	Tartrate-resistant acid phosphatase	At room temperature 8 h
		It can be stored at 2–8 °C for 3 days
FGF-23	Fibroblast growth factor 23	The sample can be stored at 2–8 °C for up to 8 h after collection
		If not measured within 8 h, the sample must be frozen

Secondly, sample collection conditions should be standardized. The sample, especially if CTX measurement is requested, should be collected between 7:30 and 10 a.m. after 8 h fasting. For NTX, second-morning urine is required. Sample storage conditions are also important. Thus, when assaying bone markers, laboratories should take into account that bone alkaline phosphatase is very stable; PINP is more stable than CTX; and osteocalcin is unstable. Potential interference from hemolysis or blood in urine (which affect osteocalcin and NTX, respectively) should also be considered. No differences have been observed between serum or plasma samples. However, CTX is more stable in plasma than in serum, and some authors recommend the use of plasma in studies that use frozen samples [15].

Finally, differences may arise based on the assay performed. Therefore, when changing to a new assay, the laboratory should compare measurements on both assays and inform the clinician about possible changes. The availability of commutable international reference materials added to the development of standard measurement procedures for the different bone markers will significantly contribute to their use in clinical practice.
